# DNA damage and cell cycle arrest induced by 2-(4-amino-3-methylphenyl)-5-fluorobenzothiazole (5F 203, NSC 703786) is attenuated in aryl hydrocarbon receptor deficient MCF-7 cells

**DOI:** 10.1038/sj.bjc.6600722

**Published:** 2003-02-18

**Authors:** V Trapani, V Patel, C-O Leong, H P Ciolino, G C Yeh, C Hose, J B Trepel, M F G Stevens, E A Sausville, A I Loaiza-Pérez

**Affiliations:** 1 School of Pharmaceutical Sciences, University of Nottingham, Nottingham NG7 2RD, UK; 2 Oral and Pharyngeal Cancer Branch, National Institutes of Dental and Craniofacial Research, Bethesda, MD, USA; 3 Cellular Defense and Carcinogenesis Section, Basic Research Laboratory, Center for Cancer Research, National Cancer Institute at Frederick, National Institutes of Health, Frederick, MD 21702-1201, USA; 4 Developmental Therapeutics Program, Division of Cancer Treatment and Diagnosis, National Cancer Institute, National Institutes of Health, Frederick, MD 21702-1201, USA; 5 Medical Oncology Clinical Research Unit, Medicine Branch, National Cancer Institute, National Institutes of Health, MD 20892, USA; 6 Developmental Therapeutics Program, Division of Cancer Treatment and Diagnosis, National Cancer Institute, National Institutes of Health, 9000 Rockville Pike, Building 10, Room 6N115, Bethesda, MD 20892, USA

**Keywords:** 2-(4-aminophenyl)benzothiazoles, aryl hydrocarbon receptor, CYP1A1, DNA damage, S-phase arrest, MCF-7

## Abstract

The fluorinated benzothiazole analogue 2-(4-amino-3-methylphenyl)-5-fluorobenzothiazole (5F 203, NSC 703786) is a novel agent with potent and selective antitumour properties and, in the form of its L-lysylamide prodrug Phortress (NSC 710305), is a current candidate for early phase clinical studies. Previous findings have indicated that cytochrome P450 1A1 (CYP1A1) may play a role in the antitumour activity of molecules in the benzothiazole series including the nonfluorinated parent compound 2-(4-amino-3-methylphenyl)benzothiazole (DF 203, NSC 674495) (Kashiyama *et al*, 1999; Chua *et al*, 2000; Loaiza-Pérez *et al*, 2002). In this study, we assessed and verified that a fully functional aryl hydrocarbon receptor (AhR) signalling pathway is a necessary requisite for the induction of efficient cytotoxicity by 5F 203 in MCF-7 wild-type sensitive cells. Drug exposure caused MCF-7 sensitive cells to arrest in G_1_ and S phase, and induced DNA adduct formation, in contrast to AhR-deficient AH^R100^ variant MCF-7 cells. In sensitive MCF-7 cells, induction of *CYP1A1* and *CYP1B1* transcription (measured by luciferase reporter assay and real-time reverse transcriptase-polymerase chain reaction (RT–PCR)), and 7-ethoxyresorufin-*O*-deethylase (EROD) activity was demonstrated, following treatment with 5F 203. In contrast, in resistant AH^R100^ cells, drug treatment did not affect *CYP1A1* and *CYP1B1* transcription and EROD activity. Furthermore, AH^R100^ cells failed to produce either protein/DNA complexes on the xenobiotic responsive element (XRE) sequence of *CYP1A1* promoter (measured by electrophoretic mobility shift assay) or DNA adducts. The data confirm that activation of the AhR signalling pathway is an important feature of the antitumour activity of 5F 203.

2-(4-Amino-3-methylphenyl)benzothiazoles are novel compounds with potent and unique antitumour properties (Shi *et al*, 1996; Bradshaw *et al*, 1998a, 1998b; Kashiyama *et al*, 1999). It was demonstrated that selective metabolism *in vitro* of the parent agent DF 203 (NSC 674495) correlated very highly with its antiproliferative activity, with uptake and biotransformation observed only in those cell lines that are sensitive to the compound, such as MCF-7 and T47D breast carcinoma cells (Kashiyama *et al*, 1999). CYP1A1, whose expression and activity are induced only in sensitive cells, appears to be responsible for the metabolic hydroxylation of DF 203 in position 6, which produces an inactive and antagonistic molecule (Chua *et al*, 2000). CYP1A1 is also postulated to have a crucial role in mediating the antitumour activity of DF 203, possibly generating an electrophilic intermediate responsible for the formation of DNA adducts in sensitive cells (Stevens *et al*, 2001).

Fluorinated derivatives of the parent drug DF 203 (see Figure 1

**Figure 1:**
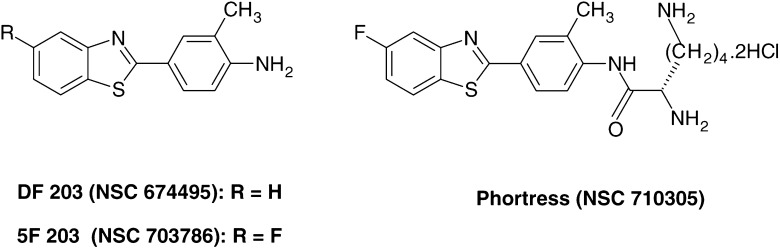
Chemical structures of antitumour 2-(4-amino-3-methylphenyl)benzothiazoles.

for structures) were synthesised in order to prevent deactivation resulting from metabolic ring hydroxylation (Hutchinson *et al*, 2001). Fluoro-analogues retain potency and selectivity, and successfully reduce or abolish the production of inactive and antagonistic metabolites and the consequent biphasic dose–response phenomenon. Drug-induced induction of CYP1A1, a crucial event in determining the antitumour specificity of this series of benzothiazoles (Chua *et al*, 2000), was not compromised by fluorination (Hutchinson *et al*, 2001).

2-(4-Amino-3-methylphenyl)-5-fluorobenzothiazole (5F 203, NSC 703786) has emerged as the most potent of the new generation of antitumour benzothiazoles both *in vitro* and *in vivo* and is currently the focus of pharmaceutical and preclinical development, as it can be converted to a readily soluble prodrug with appropriate pharmaceutical properties (Bradshaw *et al*, 2002).

As for the parent drug DF 203, depletion of 5F 203 from culture media and subsequent induction of CYP1A1 correlate with cell responsiveness. Only in sensitive cells does drug uptake occur and CYP1A1 enzyme expression increase to detectable levels; naturally occurring drug-resistant cells do not show significant changes in either drug levels in medium or CYP1A1 expression (Brantley *et al*, 2001).

We have previously reported that the parent compound DF 203 induces activation of the AhR in sensitive cells such as human breast epithelial cancer MCF-7 cells. In contrast, nonresponsive cells, for example, breast carcinoma MDA-MB-435 or prostate carcinoma PC-3 cells, showed no activation of the AhR and no induction of CYP1A1 after drug treatment (Loaiza-Pérez *et al*, 2002). We also have reported a suitable model to investigate the role of the AhR in mediating drug cytotoxicity (Loaiza-Pérez *et al*, 2001), consisting of AhR-deficient AH^R100^ cells, derived from MCF-7 human breast epithelial cancer cells by continuous exposure to escalating concentrations of benzo[*a*]pyrene (Yeh *et al*, 2001). AH^R100^ cells display relative resistance to the cytotoxic effects of several polycyclic aromatic hydrocarbons (PAHs), such as benzo[*a*]pyrene and dimethylbenz[*a*]anthracene (DMBA), due to impairment of the AhR signal transduction pathway (Yeh *et al*, 2001; Ciolino *et al*, 2002).

We therefore have used wild-type MCF-7 and AH^R100^ cells as a model, to investigate the molecular determinants for sensitivity to the clinical candidate 5F 203, including toxicity, cell cycle distribution, DNA damage, CYP1A1 activity and AhR signalling pathway activation. The results presented in this paper confirm that activation of the AhR plays an essential role in 5F 203 antitumour activity, mediating responsiveness and efficacy.

## MATERIALS AND METHODS

### Drug and cell culture

5F 203 was synthesised by the Cancer Research Laboratories at the University of Nottingham, UK and the Drug Synthesis and Chemistry Branch, NCI, following published methods (Hutchinson *et al*, 2001). The compound was dissolved in DMSO to make a 100 mM stock concentration and further diluted to the working concentration (1 nM–100 *μ*M) for experimental procedures. 2,3,7,8-Tetrachlorodibenzo-*p*-dioxin (TCDD) was prepared as 10 *μ*M top stock dissolved in DMSO and diluted to the working concentration (10 nM) immediately prior to use. MCF-7 cells were obtained from the National Cancer Institute Repository (National Cancer Institute-Frederick Cancer Research and Development Center, Frederick, MD, USA) and grown in RPMI 1640 medium supplemented with 10% fetal bovine serum (FBS) (Invitrogen, Carlsbad, CA, USA). AhR-deficient AH^R100^ cells were generated from wild-type MCF-7 cells by 6–9-month exposure to benzo[*a*]pyrene and subsequently demonstrated a 100-fold higher resistance to benzo[*a*]pyrene than the wild-type line. Additional characterisation indicated that AH^R100^ cells contained reduced amounts of AhR and normal levels of the aryl hydrocarbon receptor nuclear translocator (ARNT) (Ciolino *et al*, 2002).

### Assessment of cytotoxicity

Cells were inoculated in 96-well plates and maintained for 24 h before treatment with 5F 203, which consisted of five serial dilutions (1 : 10) ranging from 10 nM to 100 *μ*M. Treated cells were subsequently maintained for an additional 48 h, and cellular protein was measured by the sulphforhodamine-B (SRB) assay as described previously (Monks *et al*, 1991). Briefly, cells were fixed with trichloroacetic acid and stained with SRB. Protein-bound SRB was solubilised and measured spectrophotometrically to determine relative cell viability in treated and untreated cells.

### 4,6-Diamidino-2-phenylindole (DAPI) staining

Approximately 2×10^5^ cells were grown on coverslips overnight. Cells were exposed to DMSO (0.1%) or 5F 203 (1 *μ*M) for 24 h. Floating cells contained in the supernatant were collected by cytocentrifugation, and stained with 0.4% DAPI. Experiments were repeated at least three times. Stained cells were then visualised on a Zeiss Axiovert microscope using ×63 objective and images were captured with an Optronics CCD camera (Loaiza-Pérez *et al*, 2002).

### ^32^P-postlabelling assay

Pellets of control and treated (0.1% DMSO or 1 *μ*M 5F 203 for 24 h) MCF-7 and AH^R100^ cells were used for DNA extraction using Qiagen DNA extraction columns according to the manufacturer's protocol. Of each extracted DNA 5 *μ*g was digested to deoxynucleoside 3′-monophosphates by incubating with micrococcal nuclease (175 mU) and calf-spleen phosphodiesterase (6 mU) at 37°C overnight. DNA adduct enrichment was carried out by butanol extraction (Gupta, 1985). Adducts were then radiolabelled by 5′-phosphorylation using 62.5 *μ*Ci of [*γ*-^32^P]ATP and 5 U of T4 polynucleotide kinase.

### HPLC detection and quantification of DNA adducts

^32^P-labelled products were separated on a Hypersil BDS C18 analytical column (250×4.6 mm, 5 *μ*m; Shandon). The mobile phase consisted of 88% 2 M ammonium formate (pH 4.0) and 12% acetonitrile for 50 min, followed by a linear gradient of 20–45% acetonitrile for 15 min. Radioactivity was monitored by a radiochemical detector (Lab Logic, *β*-RAM) lined to a Varian Star 9012 pump. Data analysis was done by Laura, an MS Windows package (Lab Logic Inc.). The relative adduct levels (RAL) were calculated by the method of Reddy and Randerath (1986), based on the specific activity of [*γ*-^32^P]ATP and the amount of DNA used. RAL values were then translated into fmol adducts per *μ*g DNA (Gupta, 1985).

### Cell cycle analysis

Cell cycle analysis on MCF-7 and AH^R100^ cells was performed as described previously (Sherwood and Schimke, 1995). Briefly, exponentially growing cells were exposed to either DMSO (0.1%) or 5F 203 (1 *μ*M) for 24 h, then harvested, washed briefly, in cold PBS and fixed in 70% ethanol. DNA was stained by incubating the cells in PBS, containing propidium iodide (50 *μ*g ml^−1^) and RNAase A (1 mg ml^−1^) for 30 min at 37°C and fluorescence was measured and analysed using FACSCaliber (Becton Dickinson Immunocytometry Systems, San José, CA, USA) and ModFit (Verity Software, Topsham, ME, USA).

### CYP1A1 activity in intact MCF-7 and AH^R100^ cells

MCF-7 and AH^R100^ cells were grown in 24-well plates to 60–70% confluence prior to treatment with 0.1% DMSO (control) or 5F 203 (1 nM–1 *μ*M). At the end of the incubation period, the medium was aspirated and the cells were washed with PBS. CYP1A1 enzyme activity was subsequently determined by 7-ethoxyresorufin-*O*-deethylase (EROD) activity in intact cells as described by Kennedy and Jones (1994). Briefly, the fluorescence of resorufin generated from the conversion of ethoxyresorufin by CYP1A1 was measured spectrophotometrically and resorufin content was derived from a standard curve.

### Real-time RT–PCR

Evaluation of *CYP1A1* and *CYP1B1* gene expression in MCF-7 and AH^R100^ cells was performed by real-time RT–PCR, using 15 cycles of PCR, primers and probes described in a previous report (Loaiza-Pérez *et al*, 2002). RNA was isolated using Qiagen kits (Qiagen, Valencia, CA, USA). PCR efficiencies were validated by means of a standard curve.

### Transfections

Cells were transfected using LipofectAMINE (Invitrogen), with 0.5 *μ*g *Renilla reniformis* luciferase (pRL-TK) (Promega, Madison, WI, USA), and 1.5 *μ*g of pTX.Dir or pT81 as described previously (Loaiza-Pérez *et al*, 2002). Luciferase activity was measured by the Dual-Luciferase Assay System (Promega, Madison, WI, USA) following the manufacturer's instructions, and transfection efficiency was monitored by the activity of the *R. reniformis* plasmid.

### Electrophoretic mobility shift assay (EMSA)

Synthetic oligonucleotides containing the AhR-binding site of the human *CYP1A1* promoter (5′-CTC CGG TCC TTC TCA CGC AAC GCC TGG GCA-3′) (Invitrogen) were used as probes. Electrophoretic mobility shift assays were performed as described previously (Loaiza-Pérez *et al*, 2002). Briefly, nuclear extracts (20 *μ*g) from control (0.1% DMSO) and treated (1 *μ*M 5F 203 for 1 h, or 10 nM TCDD for 1 h) MCF-7 and AH^R100^ cells were incubated in binding buffer with the [^32^P]DNA probe and the resulting DNA/protein DNA complexes were separated on a 6% polyacrylamide gel (Novex) under nondenaturing conditions and high ionic strength. Gels were dried and imaged by autoradiography.

## RESULTS

### 5F 203-induced cytotoxicity correlates with AhR activation in sensitive MCF-7 cells

**Figure 2:**
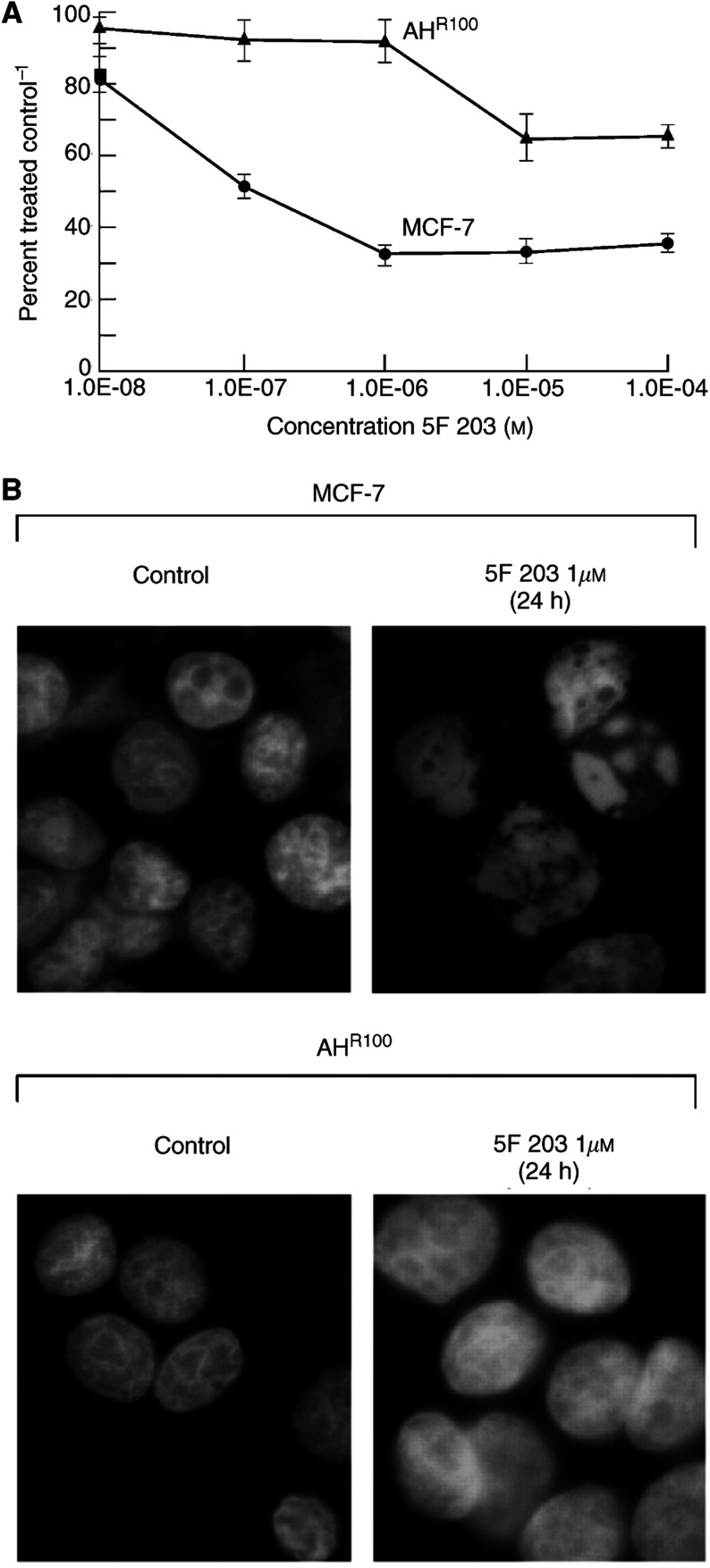
(**A**) Selective cytotoxicity of 5F 203 to MCF-7 cells. Growth inhibition induced by 5F 203 was assessed by sulphforhodamine B assay as detailed in Materials and Methods. Values are means±s.d. (*n*=10). (**B**) 5F 203 induces changes in nuclear morphology of 5F 203-sensitive MCF-7 breast cells but not AH^R100^ cells. Cells prepared as described in Materials and Methods were exposed for 24 h to 0.1% DMSO (control) or 1 *μ*M 5F 203, and stained with 4,6-diamidino-2-phenylindole (DAPI).

Previous observations have shown that cytotoxicity of benzothiazoles is mediated via activation of the AhR signalling pathway (Kashiyama *et al*, 1999; Chua *et al*, 2000; Loaiza-Pérez *et al*, 2002). In addition, cytotoxicity across a large panel of human tumour cell lines correlates with CYP1A1 inducibility (Hose *et al*, 2001). We then sought to investigate responsiveness of MCF-7 wild-type cells to the clinical candidate 5F 203 as compared to that of MCF-7-derived AhR-deficient cells (AH^R100^). Specifically, we sought to establish whether AhR-mediated signalling could account for all the cytotoxic potential of this agent. Figure 2A

indicates that MCF-7 cells are sensitive to this compound (GI_50_=121 nM, 48 h exposure). In contrast, AH^R100^ cells show a decreased growth inhibition after treatment with high drug concentration (GI_50_>100 *μ*M) and can therefore be considered resistant.

As a complementary approach, MCF-7 and AH^R100^ cells treated with 0.1% DMSO (control) or 5F 203 (1 *μ*M) for 1 h were subsequently stained by DAPI (Figure 2B). Only MCF-7 cells treated with the drug showed altered nuclear morphology, which included chromatin condensation and marginalisation to the nuclear membrane. AH^R100^ cells failed to show any significant morphological changes. Taken together, Figure 2A and 2B suggests that activation of the AhR signalling pathway participates in the antiproliferative activity of 5F 203, which results in growth inhibition and modified nuclear features.

### 5F 203 induces DNA adduct formation in MCF-7 cells

**Figure 3:**
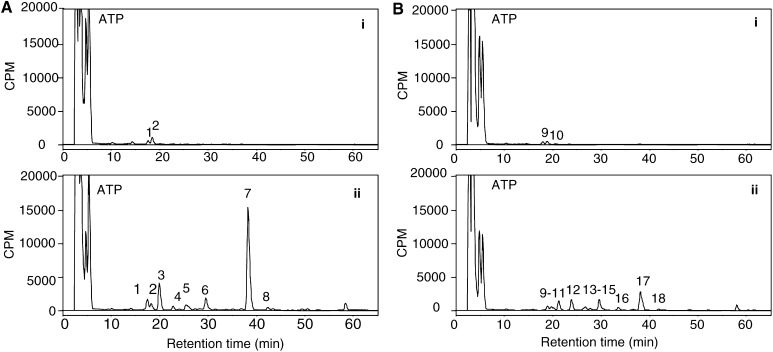
DNA adduct profiles in (**A**) MCF-7 and (**B**) AH^R100^ cells. Cells were incubated with (i) 0.1% DMSO or (ii) 1 *μ*M 5F 203 for 24 h. ^32^P-labelled adducts were analysed using 2 *μ*g digested DNA on a Hypersil C18 column with a gradient of acetonitrile in 2 M ammonium formate as detailed in Materials and Methods. The early peak is due to the presence of free [*γ*-^32^P]ATP.

We next questioned whether DNA adducts could be produced in MCF-7 and AhR-deficient AH^R100^ cells. DMSO-treated MCF-7 and AH^R100^ cells showed a similar profile of DNA adducts, with a few adducts eluting within the early retention time (⩽20 min) (Figure 3

). After treatment with 1 *μ*M 5F 203, no significant changes in these peaks were found in either cell lines, while several other adducts with longer retention time were detected. The major adduct formed in AH^R100^ cells (adduct 17 in Figure 3Bii) is chromatographically equivalent to an adduct detected in MCF-7 cells (adduct 7 in Figure 3Aii). Although DNA adduct profiles appear similar in the two cell lines, the number of total adducts/10^8^ nucleotides induced by drug treatment was greatly decreased in AH^R100^ cells compared to wild-type MCF-7 cells. These findings suggest that impairment of the AhR signalling pathway may result in reduced activation of 5F 203 into reactive species able to damage DNA.

### 5F 203 causes altered cell cycle distribution

**Figure 4:**
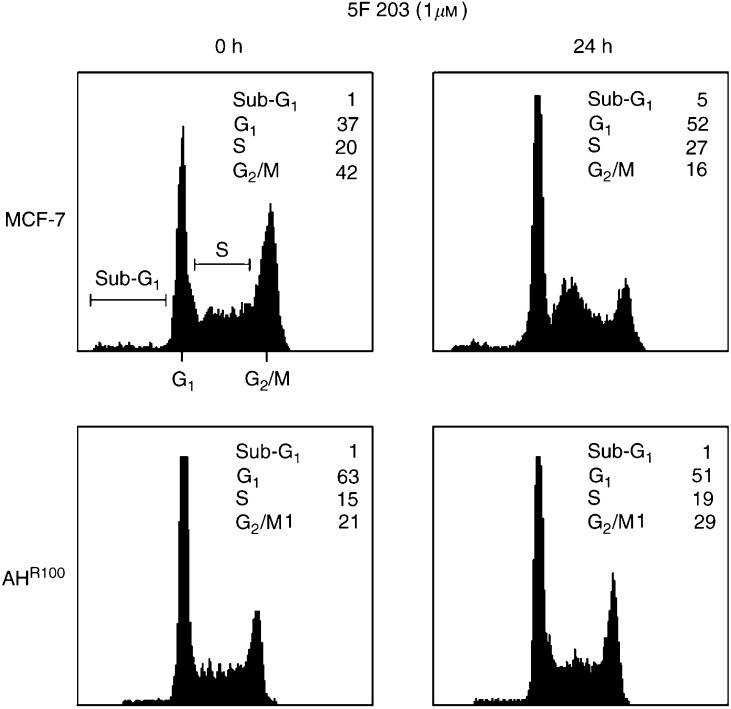
5F 203 induces accumulation of cells in S phase. Exponentially growing cells (MCF-7 and AH^R100^) were exposed to either 0.1% DMSO (control) or 5F 203 (1 *μ*M) for 24 h, harvested, washed in PBS, and fixed in 70% ethanol. DNA was stained by incubating the cells in PBS containing propidium iodide and fluorescence measured and analysed as described in Materials and Methods.

As our results in Figure 2 and Figure 3 indicated that 5F 203 was inducing the formation of DNA adducts, we investigated whether this could result in an altered cell cycle profile. For this approach, cells sensitive (MCF-7) and insensitive (AH^R100^) to 5F 203 were exposed to 1 *μ*M of the drug or 0.1% DMSO for 24 h and subsequently processed for cell cycle analysis. As illustrated in Figure 4

, 5F 203 treatment of MCF-7 cells caused a notable increase in G_1_ (37 to 52%) and S (20 to 27%) phase cells, which coincided with a decrease in G_2_/M (42 to 16%) phase cells. Accumulation of cells in sub-G_1_ was minimal when compared to control. In contrast, 5F 203-insensitive cells (AH^R100^) failed to demonstrate any notable effect on the cell cycle, in particular S, G_2_/M and sub-G_1_ phases. The data demonstrate that 5F 203-induced DNA damage may lead to cell accumulation in G_1_ and S phase concomitant with growth inhibition. As MCF-7 cells are p53 wild type, their response to 5F 203 treatment is consistent with the operation of a G_1_- and possibly S-phase checkpoint to cell cycle progression after DNA adduct formation.

### CYP1A1 and CYP1B1 induction 

**Figure 5:**
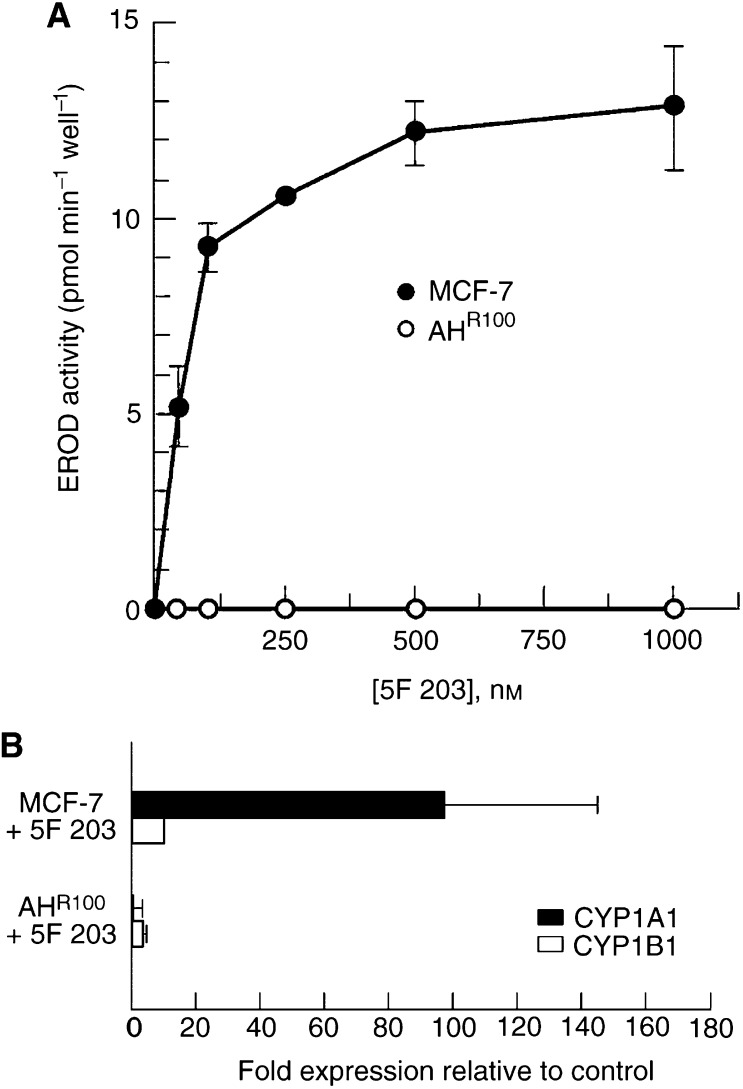
(**A**) 5F 203 induces CYP1A1 activity in MCF-7 but not in AH^R100^ cells. MCF-7 and AHR100 cells were incubated for 24 h with DMSO (0.1%) and 5F 203 (1 *μ*M) for 24 h and assayed for CYP1A1 enzyme activity by EROD assay as described in Materials and Methods. *n*=4±s.d. (**B**) 5F 203 induces *CYP1A1* and *CYP1B1* mRNA levels in sensitive (MCF-7) cells. MCF-7 and AH^R100^ cells were treated with 5F 203 (1 *μ*M) for 24 h, RNA was isolated from control and treated samples and *CYP1A1* and *CYP1B1* gene expression were measured by real-time RT–PCR as described in Materials and Methods. Data are shown as fold induction of treated cells relative to constitutive expression in control cells (±s.d., *n*=7 samples from two independent experiments).

5F 203 induces DNA damage and cell cycle arrest most obviously in MCF-7 cells with a fully functional AhR signalling pathway. The hypothesis that deficient activation of CYP1A1 might occur in AhR-deficient AH^R100^ cells was then addressed. The enzymatic activity of CYP1A1 in intact MCF-7 and AH^R100^ cells treated with 5F 203 was assayed by measuring EROD activity. Although both CYP1A1 and CYP1B1 can catalyse the dealkylation of ethoxyresorufin, the specific activity of CYP1A1 is approximately 40-fold higher than that of CYP1B1 (Doostdar *et al*, 2000). Incubation with 5F 203 for 24 h resulted in a concentration-dependent increase in EROD activity over a range of 50 nM to 1 *μ*M in MCF-7 cells, whereas the drug had no effect on EROD activity in AH^R100^ cells (Figure 5A

).

In order to compare the effect of 5F 203 on *CYP1A1* and *CYP1B1* gene expression, MCF-7 and AH^R100^ cells were treated with the compound (1 *μ*M) for 24 h and mRNA levels for these two genes were measured by real-time RT–PCR. 5F 203 caused an increase in the mRNA levels of both cytochromes in MCF-7 cells, where relative levels of *CYP1A1* mRNA were approximately 100-fold higher when compared to control; only a minimal increase of *CYP1B1* mRNA (10-fold) was observed (Figure 5B). In contrast, in AH^R100^ cells, levels of *CYP1A1* and *CYP1B1* mRNA after treatment remained similar to control (Figure 5B). These findings are in agreement with previous studies which described that the parent compound DF 203 causes induction of CYP1A1 protein and increase in mRNA levels in MCF-7 cells but not in DF 203 inherently resistant cells (Chua *et al*, 2000; Loaiza-Pérez *et al*, 2002).

### 5F 203 increases protein/DNA complex formation on the XRE sequence of CYP1A1 in MCF-7 but not AH^R100^ cells

**Figure 6:**
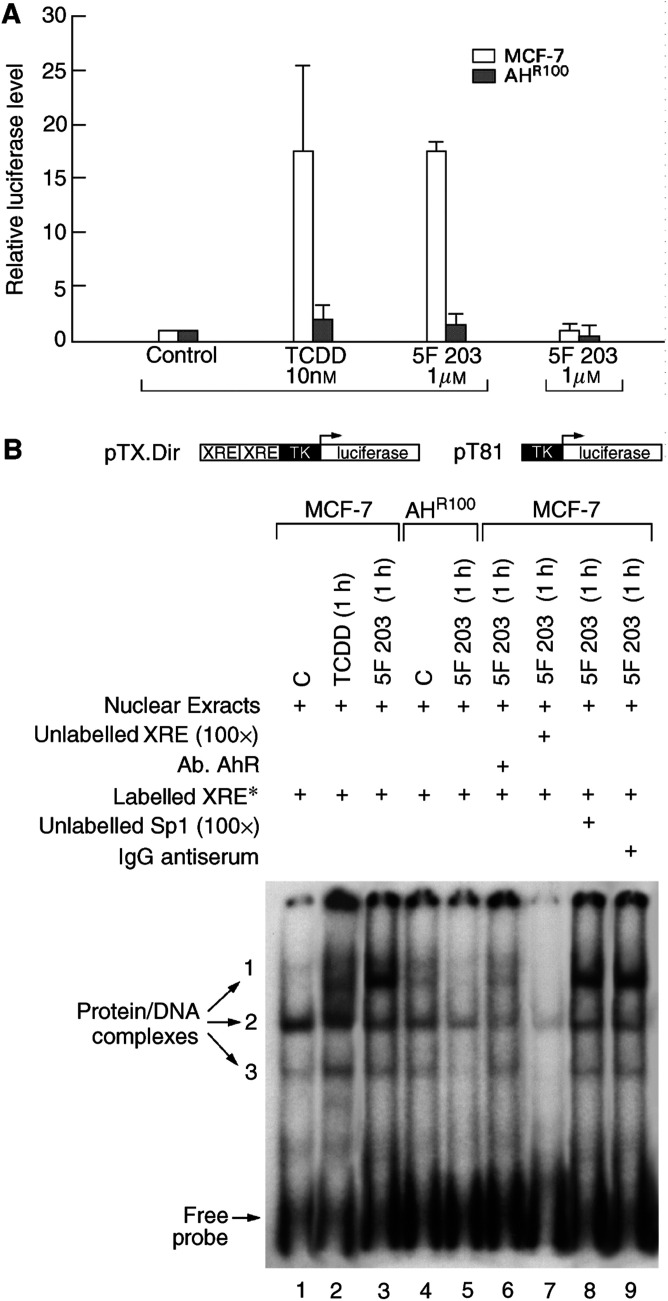
(**A**) 5F 203 induces binding to the XRE sequence of *CYP1A1*. MCF-7 and AH^R100^ cells were transfected with XRE-luciferase (pTX.Dir.) or pT81. A schematic of the respective construct is shown below the panel. Transfected cells were treated with DMSO, TCDD (10 nM) or 5F 203 (1 *μ*M) for 9 h. XRE-luciferase activity was determined normalizing to the amount of *Renilla reniformis* luciferase. The values are expressed as luciferase levels relative to control. (**B**) 5F 203 induces protein/DNA complexes on the XRE sequence of the *CYP1A1* promoter. Nuclear extracts (20 *μ*g) prepared from MCF-7 cells treated with 0.1% DMSO (C) (lane 1), TCDD (10 nM, 1 h) (lane 2) or 5F 203 (1 *μ*M, 1 h) (lane 3) were incubated with labelled XRE sequence derived from the *CYP1A1* promoter for 10 min at room temperature. Free and bound DNA were separated as described in Materials and Methods. In competition experiments, nuclear extracts from MCF-7 cells treated with 5F 203 (1 *μ*M, 1 h) were incubated with 4 *μ*g of anti-AhR antibody (lane 6), 100-fold excess of unlabelled XRE oligonucleotide (lane 7), 100-fold excess of unlabelled Sp1 oligonucleotide (lane 8) or 4 *μ*g of IgG antiserum (lane 9). Protein/DNA complexes from AH^R100^ cells were resolved in the same gel. Nuclear extracts from these cells (20 *μ*g) treated with DMSO (lane 4) or 5F 203 (1 *μ*M, 1 h) (lane 5) were incubated with radioactive XRE and resolved by the same procedure.

*CYP1A1* and *CYP1B1* promoters are regulated by the AhR, which forms a heterodimer with ARNT. Binding of the complete dimer to the XREs in the promoter region mediates transcription of AhR-responsive genes, including *CYP1A1* and *CYP1B1* (Whitlock, 1999). MCF-7 and AH^R100^ cells were transfected with an XRE-luciferase reporter construct (pTX.Dir); as control, the same reporter construct but without the XRE (pT81) was used. Cells were then treated with DMSO (0.1%), TCDD (10 nM) or 5F 203 (1 *μ*M). TCDD was used as a prototypic compound activator of *CYP1A1* transcription and included as a positive control. As shown in Figure 6A

, in MCF-7 cells transfected with pTX.Dir, a 15-fold induction of luciferase activity was observed with TCDD, and 5F 203 treatment similarly resulted in a 15-fold induction. However, when AH^R100^ cells were transfected with XRE and treated with 5F 203 (1 *μ*M), XRE-luciferase activity was demonstrated to be only approximately 1.7-fold higher than control. No induction in luciferase activity was observed when cells transfected with pT81 were treated with 5F 203 (1 *μ*M) or TCDD (10 nM). Similar results were obtained when cells were transfected with a fragment of mouse native *CYP1A1* promoter (inclusive of four dioxin responsive elements (DREs)) (pGudLuc1.1) (data not shown) (Garrison *et al*, 1996). When pTX.Dir-transfected cells were pretreated with an antagonist of AhR, *α*-naphthoflavone, before treatment with 5F 203, luciferase activity was reduced approximately 25%, suggesting competition between the two ligands for binding to the AhR (results not shown).

In order to compare protein/DNA complex formation on the XRE of *CYP1A1* promoter after treatment with 5F 203 in MCF-7 and AH^R100^ cells, electrophoretic mobility shift assays were performed. Previous reports from our laboratory (Loaiza-Pérez *et al*, 2002) described that, in MCF-7 cells, three protein/DNA complexes were found in nuclear extracts of control cells, although the intensity of the uppermost complex was lower than the others (Figure 6B, lane 1). 5F 203 (1 *μ*M) treatment for 1 h (lane 3) caused a 15-fold induction of the binding of the uppermost complex. This induction exceeded that afforded by TCDD (10 nM, 1 h) treatment (lane 2). AH^R100^ cells exposed for 1 h to 5F 203 (1 *μ*M) showed considerable attenuation of induction (lane 5). When nuclear extracts from cells treated with 5F 203 (1 *μ*M) for 1 h were pretreated with AhR polyclonal antibody (lane 6) or 100× unlabelled XRE probe (lane 7), the binding of the three complexes to labelled XRE sequences was greatly diminished. These protein/DNA complexes did not disappear when nuclear extracts from 5F 203-treated cells were preincubated with 100× unlabelled SP1 oligonucleotide (lane 8) or IgG antiserum (lane 9).

## DISCUSSION

We report here that treatment with 5F 203 causes accumulation in the G_1_ and S phase, along with growth inhibition in wild-type MCF-7 cells, but it does not affect cell cycle progression of AH^R100^ cells. We also confirm the production of DNA damage in these sensitive cells (Stevens *et al*, 2001), whereas the formation of DNA adducts is significantly reduced in the resistant subclone (Figure 3). These results corroborate the view that antitumour activity of 5F 203 is highly dependent on functional AhR signalling, whose impairment results in reduced efficacy of the agent.

Activation of the AhR signalling pathway may be an important factor determining sensitivity to 5F 203 because it triggers metabolic transformation of the drug through CYP1A1 into reactive species damaging DNA. Our data show that exposure of AH^R100^ cells to 5F 203 produces markedly fewer DNA adducts, as compared to wild-type MCF-7 cells. The chemical structure of these adducts has not yet been characterised. Computational studies using frontier molecular orbital calculations point to the putative reactive species being a nitrenium ion (Dr SE O'Brien, personal communication), but further studies are required to confirm this hypothesis and identify the adducts. A more detailed description of the formation of DNA adducts induced by benzothiazoles in sensitive cells will be published separately.

MCF-7 cells have proven to be a valuable and extensively used model for studies involving the AhR and CYP1A1 expression (Dohr *et al*, 1995; Wang *et al*, 1995; Jorgensen and Autrup, 1996). Experiments reported elsewhere (Yeh *et al*, 2001) have demonstrated that resistance to aryl hydrocarbons in AH^R100^ cells is a consequence of decreased expression of the AhR, which results in impaired induction of *CYP1A1* and probably represents an adaptation to long-term culture with polycyclic aromatic hydrocarbons (PAHs). This leads to a diminished capacity to activate PAHs like DMBA, thereby decreasing the level of apoptosis caused by exposure to PAHs. Apoptosis induced by DMBA could be blocked by inhibitors of DMBA metabolism such as *α*-naphthoflavone and diosmetin. These data demonstrated that PAH resistance in these cells is mediated by changes in the signal transduction pathway which regulates *CYP1A1* expression (Ciolino *et al*, 2002). Previous reports have demonstrated that the growth-inhibitory properties of the parent compound, DF 203, were abrogated when MCF-7 cells were cotreated with an AhR antagonist like *α*-naphthoflavone (Chua *et al*, 2000). These findings suggested that activation of AhR mediates sensitivity of MCF-7 cells to benzothiazoles. The fact that adriamycin-resistant MCF-7 cells (NCI/ADR-RES cells), which are also deficient in AhR signalling (Caruso and Batist, 1999), are resistant to benzothiazoles (Chua *et al*, 2000) further supports this hypothesis.

Activation of phase I enzymes, such as CYP1A1 and CYP1B1, usually occurs as a response to promote detoxification of xenobiotics; however, metabolic enzymes may convert inert compounds into potentially harmful products. This is the case for most PAHs, including benzo[*a*]pyrene, which induces *CYP1A1* via the AhR signalling pathway and is metabolised into reactive species that cause DNA damage (Szeliga and Dipple, 1998; Burczynski and Penning, 2000) resulting in G_1_ cell cycle arrest (Vaziri and Faller, 1997). In contrast, the environmental pollutant TCDD, which is the most potent AhR agonist, induces *CYP1A1* and modulates *CYP1B1* expression in MCF-7 cells (Dohr *et al*, 1995), but is not directly genotoxic. It has been hypothesised that TCDD-induced alterations in estrogen metabolism may lead to increased generation of reactive oxygen species and consequent induction of DNA adducts (Wyde *et al*, 2001).

In addition to selective metabolism, differential modulation of AhR functions could underlie specific cytotoxicity of the agent. Activation of the AhR signalling pathway consists of several steps, including translocation, pairing with nuclear factors, transactivation of gene transcription and degradation (Rowlands and Gustaffson, 1997), each of which might be differentially regulated in different cells. Other proteins, which integrate the function of the AhR, like heat-shock protein 90 or ARNT, may also be related to differential sensitivity to 5F 203. For example, Qin *et al* (2001) have demonstrated in patients that there is an association of ARNT splice variants with ER-negative breast cancer, poor induction of vascular endothelial growth factor under hypoxia, and poor prognosis. Further studies are necessary to determine, in greater detail, aspects of the pathway that may be altered in 5F 203-sensitive cells.
